# Individual load–velocity measures are associated with 2,000-m rowing ergometer performance in German national rowers

**DOI:** 10.3389/fspor.2025.1688650

**Published:** 2025-10-07

**Authors:** Mats Willem Jacobs, Moritz Schumann

**Affiliations:** Department of Sports Medicine and Exercise Therapy, Chemnitz University of Technology, Chemnitz, Germany

**Keywords:** peak power, maximal strength, strength testing, Olympic rowing, elite athletes

## Abstract

**Objective:**

Neuromuscular abilities were previously linked to 2,000 m rowing performance (TT) but the relationship with load-velocity profiles [LVP; e.g., peak power (PP) and power at different relative loads] remains unclear. This cross-sectional study assessed the associations between these parameters and TT and different race splits in well-trained rowers.

**Methods:**

We included 63 male (age: 18.5 ± 3.3 years) and 50 female (age: 19.3 ± 3.5 years) rowers. Within 2 weeks, 1 repetition maximum (RM), PP and power at 30% to 90% 1RM in the squat, deadlift and bench-pull were assessed by LVP and TT was performed on a Concept2 ergometer. Associations between neuromuscular parameters and TT were analyzed using generalized linear models.

**Results:**

Associations with TT were found for 1 RM in squat, deadlift and bench-pull (*β*: −32.64 to −95.15; all *p* ≤ 0.050), PP of deadlift and bench-pull (*β*: −21.79 to −71.78; *p* ≤ 0.020) but not PP of squat (*p* > 0.050). Power at 30% and 50% in squat, 30%, 50% and 70% in deadlift, and 30%, 50%, 70% and 90% in bench-pull of the respective 1 RM correlated with TT (*β*: −10.78 to −51.57; *p* < 0.050). 1 RM and PP of deadlift and bench-pull were associated with all four race splits (*p* ≤ 0.037), while PP of squat was linked only to the first 500 m (*p* < 0.030).

**Conclusion:**

While deadlift and bench-pull power appeared to be good predictors of TT, the squat power might affect TT only at low loads (i.e., 30% and 50%) and the first part of the race. These findings underline the overlooked importance of high movement velocities for rowing performance.

## 1 Introduction

Olympic rowing involves a complex interplay of physiological and neuromuscular demands, with the maximal oxygen consumption (VO_2_max) being reported as a key performance predictor (1, 2). However, during a typical rowing race, athletes maintain an average power output of 350–450 watts (W), with peaks of 600–700 watts (W) during the start spurt (3). Furthermore, stroke velocities (measured at the oar) range from 2.0 to 2.2 m·s^−1^, reaching up to 3.0–4.0 m·s^−1^ in the initial phase of the race (3). Previous research has consistently demonstrated associations between strength measures and 2,000 m rowing performance across different age groups and skill levels (4–7). Notably, particularly strong correlations have been reported between maximal strength in leg press and rowing ergometer performance (8, 9), as well as between bench pull maximal strength and rowing performance, in male and female collegiate rowers (4, 9, 10). Similar relationships have also been observed in elite female rowers for maximal dynamic strength measures such as the back squat, bench pull, and deadlift (7).

However, the required relatively high stroke velocities may indicate that generating rapid muscle contractions could also play an important role in rowing performance. This is further emphasized by different race phases consisting of a fast start (first 500 m), a steady middle section, and an intense finish (final 500 m) (11), where rowers must be capable of adjusting the force (start spurt: 1,000–1,500 N, race phase: 500–700 N, final spurt: 600–700 N) and stroke velocities (start spurt: 3.0–4.0 m·s^−1^, race phase: 2.0–2.2 m·s^−1^, final spurt: 2.2–2.8 m·s^−1^) (3). In line with this, preliminary studies have reported moderate to strong correlations (*r* = 0.3–0.9) between explosive strength measures [e.g., squat jump (SJ) and countermovement jump (CMJ) performance] and rowing ergometer performance in national junior rowers (5, 12, 13). Moreover, we previously found that the rate of force development (RFD) in leg press and midthigh pull also correlates with ergometer performance in Swiss national junior rowers (6).

To capture a range of different neuromuscular capacities [i.e., high movement velocities (MV) with low loads and low MV with high loads], load-velocity profiles (LVP) are commonly determined (14) and have been used in numerous athletic populations (15). While LVPs may clearly offer valuable insights into the specific strength capacities across varying MVs and distinct phases of the rowing race, to date only a few studies have assessed their relevance to rowing performance (5, 14). Pérez-Castilla et al. demonstrated correlations between the calculated peak velocity obtained in LVP and rowing ergometer performance in nationally and internationally competing rowers. Similarly, Giroux et al. found significant correlations between LVP-derived measures [i.e., maximal force and peak power (PP) of bench pull and SJ] and ergometer performance in national junior rowers. However, these studies primarily used LVPs to assess maximal mechanical, neuromuscular, and morphological muscle capacities in rowers (5, 14). Evidence of the relationship between neuromuscular performance at varying relative loads (e.g., power and MV) and rowing performance remains scarce. Thus, assessing neuromuscular capacities across the full LVP may significantly improve our understanding of how specific strength characteristics relate to different phases of rowing ergometer performance.

Therefore, this study primarily aimed to investigate possible associations between variables of the LVP [one repetition maximum (1 RM), PP and power at different relative loads] with the rowing ergometer performance of German national and international rowers. The secondary aim was to specifically analyze the associations of the neuromuscular abilities during the split times of the 2,000 m rowing time trial (0 m–500 m, 500 m–1,000 m, 1,000 m–1,500 m, 1,500 m–2,000 m).

## 2 Materials and methods

### 2.1 Participants

In total, 63 male (age: 18.5 ± 3.3 years, body height: 190 ± 6.8 cm, body mass: 86.3 ± 8.0 kg) and 50 female (age: 19.3 ± 3.5, body height: 176.9 ± 5.4 cm, body mass: 73.6 ± 7.8 kg) nationally and internationally competing athletes of the U17 (20 male and 10 female rowers), U19 (21 male and 14 female rowers), U23 (9 male and 20 female rowers) and Elite (those older than 23 years) (13 male and 6 female rowers) category participated in this study. Inclusion criteria were as follows: 1) participation in at least national-level rowing competitions, 2) regular strength training for at least one year, and 3) absence of injuries or illnesses affecting performance. Athletes in the U17 and U19 categories were classified as highly trained/national level (Tier 3), U23 athletes as elite/international level (Tier 4), and Elite category athletes as world class (Tier 5) (16). All procedures were carried out after the participants provided written informed consent. The study was approved by the local ethics committee (#101676079) and conducted according to the Declaration of Helsinki.

### 2.2 Design

**Figure 1:**
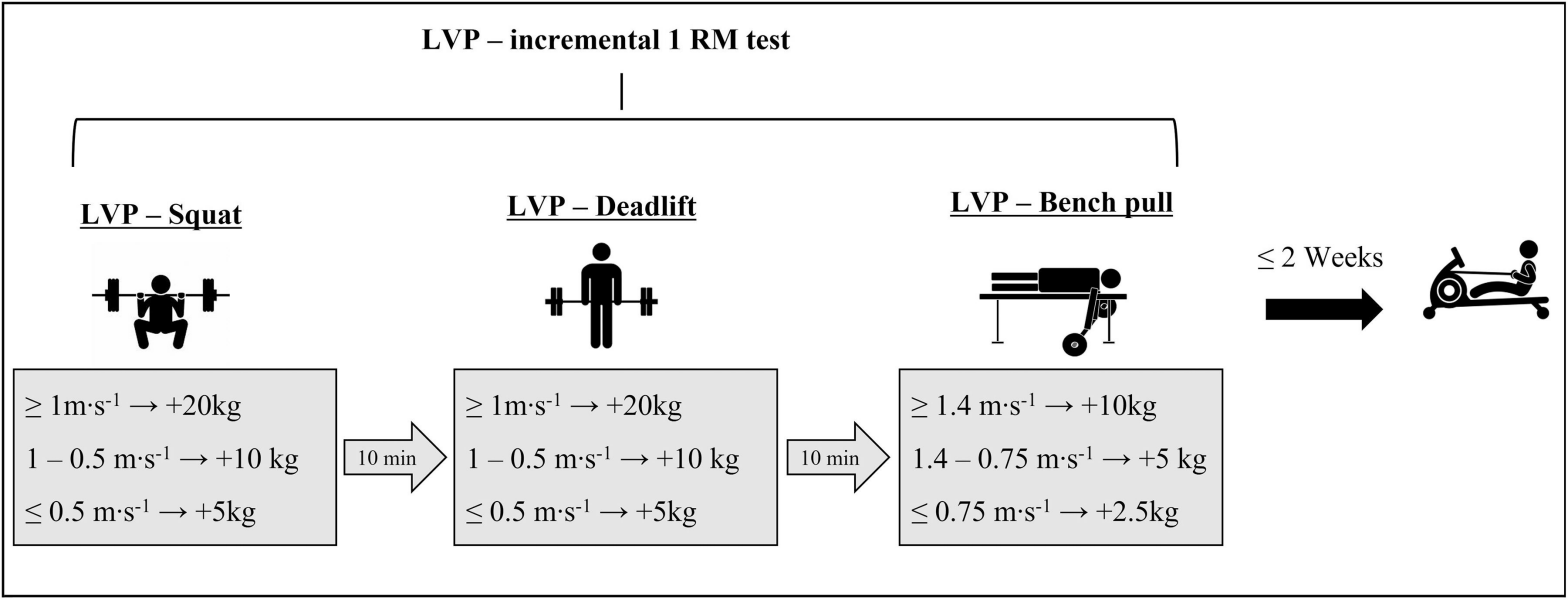
Study design showing the incremental 1 RM test for the three strength exercises squat, deadlift and bench pull as well as the 2,000 m ergometer test two weeks afterwards. Furthermore, the velocity thresholds which defined the increments of the 1 RM test are shown for each particular exercise.

The testing protocol consisted of assessing an individual LVP for three exercises (i.e., squat, deadlift and bench pull), covering the main muscle groups involved in Olympic rowing. Additionally, within two weeks following the LVP, a 2,000 m rowing ergometer time trial was performed (Figure 1). The athletes were instructed to refrain from intense training sessions within 24 h prior to the testing to ensure maximal performance.

### 2.3 Load-velocity assessment

Before the LVP assessment, athletes completed a standardized 15-min warm-up: 10 min of light rowing on the ergometer followed by 5 min of individual mobility exercises. Prior to each LVP assessment, the male athletes performed an exercise-specific warm-up of 10 repetitions with loads of 30 kg (squat), 40 kg (deadlift), and 30 kg (bench pull) and the female athletes' warm-up consisted of 20 kg (squat), 30 kg (deadlift) and 20 kg (bench pull). The LVPs were assessed by incremental 1 RM tests in squat, deadlift, and bench pull, with 5–10 increments and 2 min rest between sets. At least 5 data points (squat: 6.9 ± 1.5, deadlift: 6.8 ± 1.5, bench pull: 5.6 ± 1.5) were collected per test to reliably calculate MV and power curves. The protocol, adapted from Sanchez-Medina et al. (17), used velocity thresholds to adjust loads and repetitions (Figure 1):

• Squat/deadlift: MV > 1.0 m·s^−1^ = +20 kg (3 repetitions), 1.0–0.5 m·s^−1^ = +10 kg (2 repetitions), MV < 0.5 m·s^−1^ = +5 kg (1 repetition)
• Bench pull: MV > 1.5 m·s^−1^ = +10 kg (3 repetitions), 1.5–1.0 m·s^−1^ = +5 kg (2 repetitions), MV < 0.75 m·s^−1^ = +2.5 kg (1 repetition)

The MV was assessed for each repetition by a linear velocity transducer (Gymaware RS, Kinetic Performance, Braddon ACT 2612 Australia), known for high validity and reliability (18). The LVP assessment was terminated when the athletes could not fully lift the load without external assistance. All athletes completed the exercises in the same order, starting with squat, followed by deadlift and bench pull.

Squat depth was individually standardized using the mean “dip” (i.e., eccentric movement range) from the 10 warm-up repetitions ±10%. If the athletes failed to reach the defined squat depth, the respective repetition was repeated. To minimize the effects of the stretch-shortening cycle, the eccentric movement was controlled; a pause of approximately 0.5–1 s was performed at the reversal point and an acoustic signal initiated the concentric contraction. The LVPs for the deadlift and bench pull followed the same protocol as the squat, with the key difference being that the barbell started from a stationary position on the floor. Athletes were instructed to build up tension before initiating the concentric phase upon an acoustic signal. The bench pull was done on a customized bench adjusted to arm length; arms had to be fully extended at the start, and a rep counted when the barbell touched the bench. A repetition in squat and deadlift was counted when the athletes carried out a full concentric movement without external help.

The 1 RM was defined as the highest load lifted independently by the athletes. The MV was extracted as mean velocity [m·s^−1^]. If multiple repetitions were completed in a set, the fastest velocity was used for further analysis. The power [W] was calculated as follows: (MV [m·s^−1^] · load [kg]) · 9.81 m·s^−1^). Afterwards, the individual MV was fitted to a linear function and the power was fitted to a second-degree polynomial function. The respective function was then used for further analysis of the MV and power variables. The PP [W] was calculated using the maximum of the respective polynomial function. These functions were used to estimate MV and power at 30%, 50%, 70%, and 90% of 1 RM as conducted in previous studies (19) and, therefore, gain insight into the MV and power at different relative loads.

### 2.4 Rowing performance testing

A 2,000 m rowing ergometer test was conducted according to the German Rowing Association's guidelines. Room temperature was maintained between 18 and 22 °C with humidity below 70%. The test was performed on a Concept2 rowing ergometer (Concept2 Deutschland GmbH, 22041 Hamburg, Germany) using a drag factor of 145 for male and 130 for female athletes. Stroke rate was self-selected. Prior to testing, athletes completed a 10–15-min individualized warm-up, including low-intensity rowing and mobility exercises. Performance metrics included total time, average power, and split times for each 500 m segment.

### 2.5 Statistical analysis

Statistical analyses were performed using R Studio (v4.4.2, Posit PBC, Boston, USA). Normality of the data distribution was analyzed using the Shapiro–Wilk test (package: stats). Normal distribution was confirmed for 1 RM and PP values across all three exercises in male athletes, and for 1 RM values in female athletes. Accordingly, one-way ANOVA (stats package) was used to assess differences in 1 RM and PP across age categories, with Bonferroni-corrected *post hoc* tests applied when significant group effects were found. For PP values in female athletes, which were not normally distributed, a Kruskal–Wallis test (rstatix package) was conducted, followed by a Bonferroni-adjusted Dunn *post hoc* test when applicable. Due to non-normal distribution in some neuromuscular parameters, generalized linear models (GLMs; stats package) were used to evaluate associations between strength variables (i.e., PP, 1 RM, and power at various relative intensities) and rowing performance (total 2,000 m time and 500 m splits) in both sexes. Since the effect of LVP variables on rowing performance might not be linear, we assessed the most appropriate function between the neuromuscular parameters and rowing ergometer performance by fitting four generalized linear models: a linear, a quadratic, a logarithmic (log-transformed predictor), and an exponential model (log-transformed outcome). All models were evaluated using 10-fold cross-validation (package: caret, rsample). This comparison enabled us to quantify model accuracy taking into consideration the Mean absolute error (MAE), the Root Mean Square Error (RMSE) and the Coefficient of Determination (*R*^2^). The logarithmic GLMs showed the best fit (MAE: 13.38 ± 1.10, RMSE: 16.48 ± 1.40, *R*^2^: 60.71 ± 5.30%) and were *p* value adjusted using an FDR correction for multiple tests. BMI was included as a covariate to control for biological heterogeneity (e.g., body mass and height). Results are presented as mean ± SD, with significance set at *p* < 0.050.

## 3 Results

**Figure 2:**
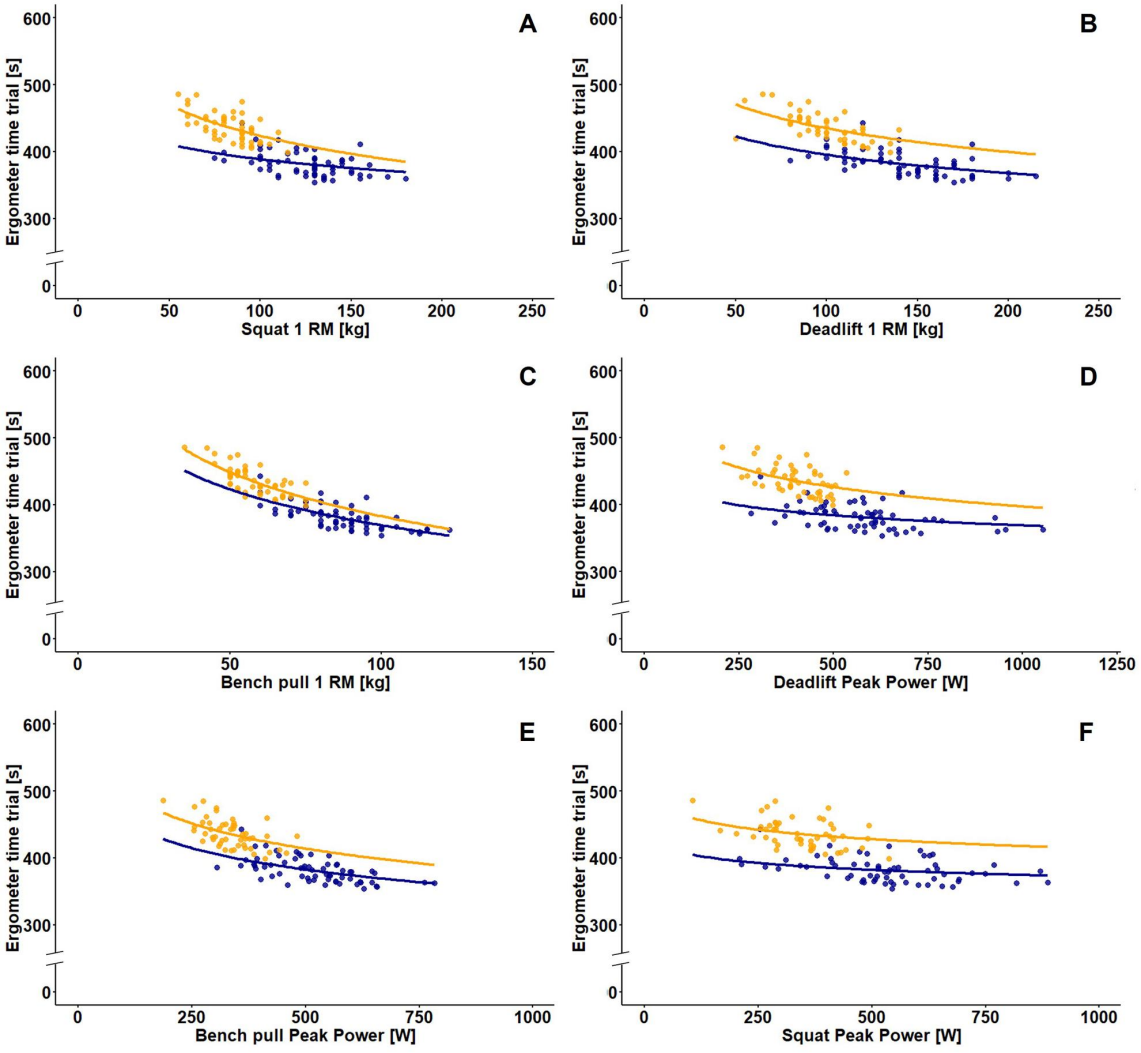
Logarithmic generalized linear model for the association between the 1 RM [kg] of squat **(A)**, deadlift **(B)** and bench pull **(C)** as well as peak power [watt (W)] of deadlift **(D)**, bench pull **(E)** and squat **(F)** with 2,000 m rowing ergometer performance(s). Data of the male athletes are indicated by the color blue and data of the female athletes by the color yellow.

The detailed descriptive data of the 1 RM and PP as well as the statistical differences between age groups is shown in Supplementary Table 1 and Figures 1, 2.

### 3.1 Associations of maximal strength and peak power with 2,000 m rowing performance

The 1 RM of the squat (male: *β*: −32.64, *p* = 0.035, [−58.93 to −6.34]/female: *β*: −66.75, *p* = 0.002, [−103.02 to −30.48]) (Figure 2A), deadlift (male: *β*: −39.95, *p* = 0.002, [−62.96 to −16.94]/female: *β*: −51.38, *p* = 0.002, [−75.24 to −27.52]) (Figure 2B) and bench pull (male: *β*: −77.63, *p* = 0.002, [−104.41 to −50.86]/female: *β*: −95.15, *p* = 0.002, [−126.60 to −63.70]) (Figure 2C) was statistically associated with the 2,000 m rowing ergometer performance. Thus, the squat 1 RM explained 31.6% of variance in the 2,000 m time in male and 57.9% in female athletes, while the deadlift 1 RM explained 45.3% in male and 70.4% in female athletes and the bench pull 1 RM 56.3% in male and 62.2% in female athletes, respectively. Associations of PP and 2,000 m rowing ergometer performance were found for deadlift (male: *β*: −21.79, *p* = 0.020, [−38.23 to −5.36]/female: *β*: −41.97, *p* = 0.020, [−71.78 to −12.16]) (Figure 2D) and bench pull (male: *β*: −46.55, *p* = 0.002, [−65.64 to −27.46]/female: *β*: −54.36, *p* = 0.009, [−84.59 to −24.14]) (Figure 2E) but not squat (male: *β*: −14.85, *p* = 0.076, [−29.79 to −0.09]/female: *β*: −20.48, *p* = 0.098, [−42.14 to 1.18]) (Figure 2F). The deadlift power thereby explained 36.7% of variance in 2,000 m time in male and 59.3% in female athletes while the bench pull power explained 43.5% in male and 55.2% in female athletes.

### 3.2 Associations of power at different relative loads with 2,000 m rowing performance

The coefficients of the power at different relative loads of the 1 RM are shown in Table 1. The power in the squat was only significantly associated with 2,000 m rowing ergometer performance in male athletes for 30% and 50% of the individual 1 RM but not in female athletes. Furthermore, the power in the deadlift at 30%, at 50% and 70% of the individual 1 RM was statistically associated with 2,000 m rowing ergometer performance in both sexes. While the power in bench pull at 30% 1 RM was only statistically associated with 2,000 m rowing ergometer performance in male athletes, the bench pull power at 50%, 70% and 90% was statistically associated with 2,000 m rowing ergometer performance in both sexes.

**Table 1:** Associations of the power at different relative loads (i.e., 30%, 50%, 70%, 90% of the 1 RM) and the 2,000 m rowing ergometer performance. The table shows the coefficient (influence on the depended variable) of the power variable and the respective confidence intervals of the three exercises.

Male					
2,000 m					
SP30	*β*: −14.87* [−27.26 to −2.48]	DP30	*β*: −26.63** [−41.15 to −12.10]	BP30	*β*: −10.78** [−18.54 to −3.03]
SP50	*β*: −16.34* [−30.48 to −2.21]	DP50	*β*: −26.85** [−42.44 to −11.25]	BP50	*β*: −40.09** [−58.19 to −21.99]
SP70	*β*: −12.36 [−26.98–2.26]	DP70	*β*: −23.09** [−39.72 to −6.47]	BP70	*β*: −40.71** [−59.94 to −21.47]
SP90	*β*: −4.24 [−18.71 to 10.24]	DP90	*β*: −11.23 [−28.02 to 5.57]	BP90	*β*: −36.43** [ −54.74 to −18.13]
Female					
2,000 m					
SP30	*β*: −17.87 [−37.12 to 1.37]	DP30	*β*: −23.36* [−42.66 to −4.07]	BP30	*β*: −7.61 [−19.16 to 3.95]
SP50	*β*: −20.24 [−39.92 to −0.56]	DP50	*β*: −40.08* [−64.87 to −15.29]	BP50	*β*: −42.85* [−74.73 to −10.97]
SP70	*β*: −18.48 [−39.93 to 2.96]	DP70	*β*: −42.27* [−71.64 to −12.90]	BP70	*β*: −51.57* [−87.27 to −15.87]
SP90	*β*: −6.71 [−32.77 to 19.34]	DP90	*β*: −17.18 [−48.41 to 14.05]	BP90	*β*: −35.03* [−64.63 to −5.43]

Bold coefficients indicate statistical associations.

SP, squat; DP, deadlift; BP, bench pull.

* *p* < 0.05. **p ≤ 0.01.

### 3.3 Associations of the 1 RM and peak power with 500 m splits

The coefficients of the association of 1 RM and PP with the 500 m splits are shown in Table 2. The 1 RM of the squat (male: *β*: −9.02 to −8.06, *p* ≤ 0.032/female: *β*: −17.64 to −15.95, *p* ≤ 0.005), deadlift (male: *β*: −10.70 to −9.36, *p* ≤ 0.037/female: *β*: −12.30 to −10.80, *p* ≤ 0.003) and bench pull (male: *β*: −20.63 to −18.67, *p* ≤ 0.002/female: *β*: −24.51 to −22.73, *p* ≤ 0.001) was (with exception of Squat 1 RM and the 3. 500 m Split in male athletes) statistically associated with all of the four 500 m splits in male and female athletes respectively. While PP in the squat was only statistically associated with the first 500 m (male: *β*: −4.08; *p* = 0.024/female: *β*: −5.77; *p* = 0.030), the PP in deadlift (male: *β*: −5.97 to −4.93, *p* ≤ 0.049/female: *β*: −12.22 to −10.02, *p* ≤ 0.018) and bench pull (male: *β*: −12.95 to −10.76, *p* ≤ 0.002/female: *β*: −14.24 to 13.35, *p* ≤ 0.003) was statistically associated with all four 500 m splits in both sexes.

**Table 2:** The table shows the coefficient (influence on the depended variable) and confidence intervals of the associations of 1 RM [kg] and PP [W] with the 500 m splits of rowing ergometer performance.

Male				
Neuromuscular capacity	0–500 m	500 m–1,000 m	1,000 m–1,500 m	1,500–2,000 m
Squat 1 RM [kg]	*β*: −8.06* [−13.95 to −2.17	*β*: −8.26* [−14.90 to −1.63]	*β*: −7.75 [−15.29 to −0.22]	*β*: −9.02* [−16.86 to −1.18]
Deadlift 1 RM [kg]	*β*: −9.36** [−14.53 to −4.19]	*β*: −10.60** [−16.35 to −4.85]	*β*: −10.70** [−17.26 to −4.13]	*β*: −10.68* [−17.61 to −3.74]
Bench pull 1 RM [kg]	*β*: −18.67** [−24.51 to −12.83]	*β*: −19.94** [−26.63 to −13.25]	*β*: −20.55** [−28.36 to −12.74]	*β*: −20.63** [−28.97 to −12.30]
Squat PP [W]	*β*: −4.08* [−7.41 to −0.75]	*β*: −3.58 [−7.36 to 0.20]	*β*: −3.02 [−7.31 to 1.26]	*β*: −4.07 [−8.51 to 0.38]
Deadlift PP [W]	*β*: −5.52** [−9.18 to −1.85]	*β*: −5.36* [−9.52 to −1.20]	*β*: −4.93* [−9.67 to −0.19]	*β*: −5.97* [−10.88 to −1.06]
Bench pull PP [W]	*β*: −10.89** [−15.16 to −6.63]	*β*: −10.76** [−15.72 to −5.80]	*β*: −12.02** [−17.58 to −6.46]	*β*: −12.95** [−18.73 to −7.17]
Female				
Neuromuscular capacity	0–500 m	500 m–1,000 m	1,000 m–1,500 m	1,500–2,000 m
Squat 1 RM [kg]	*β*: −17.46*** [−25.90 to −9.02]	*β*: −15.95*** [−25.36 to −6.55]	*β*: −16.59*** [−26.40 to −6.78]	*β*: −16.48** [−27.66 to −5.29]
Deadlift 1 RM [kg]	*β*: −10.80*** [−16.75 to −4.85]	*β*: −11.56*** [−17.90 to −5.22]	*β*: −12.30*** [−18.87 to −5.73]	*β*: −11.91** [−19.47 to −4.34]
Bench pull 1 RM [kg]	*β*: −22.73*** [−30.26 to −15.19]	*β*: −24.51*** [−32.48 to −6.55]	*β*: −24.40*** [−32.98 to −5.81]	*β*: −24.25*** [−34.43 to −4.07]
Squat PP [W]	*β*: −5.77* [−10.88 to −0.66]	*β*: −5.34 [−10.86 to 0.18]	*β*: −4.46 [−10.29 to 1.38]	*β*: −5.06 [−11.54 to 1.43]
Deadlift PP [W]	*β*: −11.72*** [−18.64 to −4.79]	*β*: −10.30** [−17.95 to −2.65]	*β*: −10.02* [−18.07 to −1.96]	*β*: −12.22** [−21.07 to −3.37]
Bench pull PP [W]	*β*: −13.35*** [−20.53 to −6.17]	*β*: −14.00*** [−21.70 to −6.31]	*β*: −13.93*** [−22.04 to −5.81]	*β*: −14.24** [−23.45 to −5.04]

Bold coefficients indicate statistical associations.

* *p* < 0.05. **p ≤ 0.01. ***p ≤ 0.001.

### 3.4 Associations of the power over the LVP with 500 m splits

Detailed results and respective coefficients and confidence intervals of all models are shown in Tables 3, 4. Squat power (SP) across the LVP spectrum showed only isolated significant associations with the 500 m split times. In male athletes, significant relationships were observed for power at 30% and 50% of LVP with the 1st, 2nd, and 4th splits, as well as for power at 70% with the 1st split (all: *p* < 0.050). In female athletes, only the model including power at 50% of LVP and the 1st split was significant (*p* < 0.050). The analysis of the relationship between deadlift power (DP) across the entire LVP and the 500 m race segments yielded significant models for DP30, DP50, and DP70 with all four race segments in male athletes (all: *p* < 0.050). In female athletes, the analysis revealed significant associations between DP30 and split segments 1 and 4, as well as between DP50 and DP70 with all four race segments (all: *p* < 0.050). The analysis of bench pull power (BP) across the entire LVP in relation to the individual 500 m race segments yielded significant models for the entire LVP with all race segments in male athletes (all: *p* < 0.050). In female athletes, significant associations were observed between BP50 and race segments 1, 3, and 4; between BP70 and all four race segments as well as BP90 and the first 500 m segment (all: *p* < 0.050).

**Table 3:** The table shows the coefficient (influence on the depended variable) and confidence intervals of the associations of the power at 30%, 50%, 70% and 90% of the three exercises with the 500 m splits of rowing ergometer performance for the male athletes.

Male				
Neuromuscular capacity	0–500 m	500 m–1,000 m	1,000 m–1,500 m	1,500–2,000 m
SP30	*β*: −3.71* [−6.49 to −0.94]	*β*: −3.58* [−6.72 to −0.44]	*β*: −3.28 [−6.84 to 0.28]	*β*: −4.29* [−7.97 to −0.61]
SP50	*β*: −4.30* [−7.45 to −1.15 ]	*β*: −3.94* [−7.52 to −0.36]	*β*: −3.44 [−7.51 to 0.63]	*β*: −4.56* [−8.77 to −0.36]
SP70	*β*: −3.57* [−6.83 to −0.31]	*β*: −3.00 [−6.70 to 0.69]	*β*: −2.37 [−6.56 to 1.81]	*β*: −3.28 [−7.63 to 1.07]
SP90	*β*: −1.95 [−5.20 to 1.29]	*β*: −1.07 [−4.72 to 2.58]	*β*: −0.34 [−4.44 to 3.76]	*β*: −0.76 [−5.06 to 3.53]
Neuromuscular capacity	0–500 m	500 m–1,000 m	1,000 m–1,500 m	1,500–2,000 m
DP30	*β*: −6.62** [−9.83 to −3.40]	*β*: −6.66** [−10.34 to −2.99]	*β*: −6.50** [−10.71 to −2.29]	*β*: −7.04** [−11.43 to −2.64]
DP50	*β*: −6.46** [−9.95 to −2.98]	*β*: −6.77** [−10.70 to −2.83]	*β*: −6.57** [−11.08 to −2.06]	*β*: −7.13** [−11.83 to −2.43]
DP70	*β*: −5.60** [−9.32 to −1.87]	*β*: −5.81* [−10.00 to −1.61]	*β*: −5.47* [−10.25 to −0.68]	*β*: −6.17* [−11.15 to −1.19]
DP90	*β*: −3.03 [−6.81 to 0.75]	*β*: −2.79 [−7.03 to 1.45]	*β*: −2.15 [−6.93 to 2.63]	*β*: −2.97 [−7.96 to 2.02]
Neuromuscular capacity	0–500 m	500 m–1,000 m	1,000 m–1,500 m	1,500–2,000 m
BP30	*β*: −2.43* [−4.19 to −0.67]	*β*: −2.85** [−4.79 to −0.90]	*β*: −2.57* [−4.80 to −0.34]	*β*: −2.97* [−5.29 to −0.66]
BP50	*β*: −9.64** [−13.66 to −5.63]	*β*: −9.66** [−14.29 to −5.03]	*β*: −9.74** [−15.06 to −4.42]	*β*: −11.08** [−16.55 to −5.61]
BP70	*β*: −9.88** [−14.14 to −5.63]	*β*: −9.42** [−14.39 to −4.46]	*β*: −10.04** [−15.66 to −4.42]	*β*: −11.36** [−17.14 to −5.57]
BP90	*β*: −8.44** [−12.55 to −4.32]	*β*: −8.41** [−13.13 to −3.70]	*β*: −9.14** [−14.45 to −3.83]	*β*: −10.44** [−15.90 to −4.97]

Bold coefficients indicate statistical associations.

SP, squat; DP, deadlift; BP, bench pull.

* *p* < 0.05. **p ≤ 0.01.

**Table 4:** The table shows the coefficient (influence on the depended variable) and confidence intervals of the associations of the power at 30%, 50%, 70% and 90% of the three exercises with the 500 m splits of rowing ergometer performance for the female athletes.

Female				
Neuromuscular capacity	0–500 m	500 m–1,000 m	1,000 m–1,500 m	1,500–2,000 m
SP30	*β*: −5.10 [−9.63 to −0.56]	*β*: −3.95 [−8.90 to 1.00]	*β*: −4.22 [−9.37 to 0.94]	*β*: −4.82 [−10.55 to 0.92]
SP50	*β*: −5.60* [−10.24 to −0.95]	*β*: −5.10 [−10.13 to −0.07]	*β*: −4.62 [−9.91 to 0.67]	*β*: −5.18 [−11.07 to 0.71]
SP70	*β*: −5.22 [−10.29 to −0.15]	*β*: −4.69 [−10.16 to 0.79]	*β*: −4.06 [−9.82 to 1.70]	*β*: −4.69 [−11.09 to 1.71]
SP90	*β*: −2.62 [−8.82 to 3.59]	*β*: −1.04 [−7.70 to 5.62]	*β*: −0.93 [−7.87 to 6.01]	*β*: −1.89 [−9.60 to 5.83]
Neuromuscular capacity	0–500 m	500 m–1,000 m	1,000 m–1,500 m	1,500–2,000 m
DP30	*β*: −6.42* [−10.95 to −1.89]	*β*: −5.59 [−10.54 to −0.63]	*β*: −5.32 [−10.53 to −0.11]	*β*: −7.33* [−13.00 to −1.66]
DP50	*β*: −10.42** [−16.24 to −4.61]	*β*: −9.81** [−16.19 to −3.43]	*β*: −9.82* [−16.52 to −3.12]	*β*: −11.42* [−18.82 to −4.02]
DP70	*β*: −11.58* [−18.42 to −4.74]	*β*: −10.38* [−17.92 to −2.84]	*β*: −10.29* [−18.21 to −2.38]	*β*: −12.05* [−20.80 to −3.30]
DP90	*β*: −6.71 [−14.03 to 0.61]	*β*: −4.07 [−12.05 to 3.92]	*β*: −3.37 [−11.73 to 4.98]	*β*: −6.12 [−15.31 to 3.08]
Neuromuscular capacity	0–500 m	500 m–1,000 m	1,000 m–1,500 m	1,500–2,000 m
BP30	*β*: −1.79 [−4.56 to 0.98]	*β*: −1.66 [−4.63 to 1.30]	*β*: −1.88 [−4.97 to 1.20]	*β*: −2.16 [−5.58 to 1.27]
BP50	*β*: −9.29* [−17.02 to −1.56 ]	*β*: −9.66 [−17.93 to −1.40]	*β*: −11.67* [−20.12 to −3.22]	*β*: −12.33* [−21.81 to −2.85]
BP70	*β*: −11.78* [−20.38 to −3.17]	*β*: −12.37* [−21.56 to −3.17]	*β*: −14.21* [−23.64 to −4.78]	*β*: −14.27* [−24.96 to −3.59]
BP90	*β*: −8.87* [−15.91 to −1.84]	*β*: −8.46 [−16.05 to −0.86]	*β*: −9.20 [−17.08 to −1.31]	*β*: −9.57 [−18.41 to −0.73]

Bold coefficients indicate statistical associations.

SP, Squat; DP, deadlift; BP, bench pull.

*p < 0.05. **p ≤ 0.01.

## 4 Discussion

We demonstrated that, in addition to the 1 RM in squat, deadlift, and bench pull, the PP of the deadlift and bench pull was also statistically associated with 2,000 m rowing ergometer performance in well-trained male and female athletes across all age groups. Furthermore, our study highlighted the importance of power production by demonstrating statistical associations between the power in the squat, deadlift and bench pull at different relative loads (squat: 30% and 50%; deadlift: 30%, 50% and 70%; bench pull: 30%, 50%, 70% and 90%) with the total 2,000 m time as well as specific 500 m split times. Finally, while the Squat PP was only associated with the first 500 m of the race, the PP of the deadlift and bench pull as well as the 1 RM of all exercises were statistically associated with all 500 m splits.

The observed associations between the 1 RM of the three exercises and the 2,000 m rowing ergometer performance observed in our study are well in line with most of the previous studies that reported correlations between either the dynamic 1 RM and rowing ergometer performance (7, 20) or isometric and isokinetic maximal strength measures and rowing performance (6, 10, 21). However, extending current knowledge, we found that bench pull PP was significantly associated with 2,000-m rowing ergometer performance across different age groups and both sexes, demonstrating its relevance to rowing performance irrespective of age or sex. Previous studies have also reported associations between bench pull PP and rowing ergometer performance (5, 14), but either focused on 1,500-m performance in junior rowers (5) or did not differentiate between male and female athletes (14). The associations between bench pull PP and rowing performance appear reasonable, as the bench pull exercise likely reflects the finish phase (i.e., boat acceleration by an arm pull, ending in the removal of the blades from the water) of the rowing stroke. This phase is characterized by the highest stroke velocity, particularly when compared to the catch (i.e., beginning of the drive, immersion of the blades into the water) and leg drive phases (i.e., initial boat acceleration, most work done by leg muscle contraction) (22). Although force output is lower in the finish phase, the higher stroke velocity suggests that the ability to produce power at high speeds is crucial, which may explain the observed relationship between bench pull PP and 2,000 m ergometer performance. While the bench pull exercise might reflect the important finish phase of the rowing stroke, it was previously suggested that especially the power transfer from the leg drive to the finish phase is crucial in the rowing stroke and might be reflected by exercises like deadlift or power clean targeting the trunk neuromuscular capacities (23). Thus, the statistical associations between deadlift PP and rowing ergometer performance found in our study might play a crucial role in explaining 2,000 m rowing ergometer performance. Our findings are supported by previous work, showing that the boat acceleration further increases during this transition phase (22), highlighting the importance of generating high power at fast MV's with the progression of the rowing stroke, potentially resulting in a reliance of PP in the deadlift.

Besides a good finish phase and transition phase especially the leg drive is crucial in the rowing stroke and accounts for approximately 50% of the force generation (24). Surprisingly, we did not find significant associations between the PP in the squat and 2,000 m rowing ergometer performance, although earlier findings already linked lower-limb power (e.g., SJ, CMJ) to rowing ergometer performance (5, 12, 13). Previous studies have shown that the velocity of the leg muscles (measured as rate of variation in the seat position) as well as the boat's acceleration at the beginning of the stroke is relatively slow (22). Thus, it might be hypothesized that absolute leg power may be less critical at the start of the stroke due to limited boat acceleration potential, while maximal strength appears more important than high-velocity power output during the leg drive phase. However, while we controlled for the technique (e.g., squat depth, pause at the reversal point) in the deep squat, individual skill levels and, therefore, the ability to generate the highest possible MVs and highest possible PP have to be considered as confounder, potentially limiting the generalizability of our results.

Interestingly, while the PP of the squat was not associated with the total 2,000 m rowing ergometer performance, we observed statistical associations of the squat power at 30% and 50% of the individual 1 RM and the 2,000 m time which was underlined by the associations with the individual 500 m split times (with the exception of the 3. 500 m split time; see Table 3). Given that the leg drive phase is typically characterized by high forces and the relatively low velocity of the legs (22), these findings were somewhat surprising. The reasons for this remain speculative and it cannot be ruled out that this was due to the high variability in technique. However, while the catch and early leg drive phases involve high force but relatively low movement velocity with the stroke progressing, boat acceleration increases, potentially requiring higher movement velocities in the later part of the leg drive. Thus, the associations with the power at high movement velocities might be influenced by the decreasing knee joint angles during the stroke which may enhance contraction efficiency, allowing for greater movement velocity and power output (25) and aligns with findings showing peak lower limb muscle activation around a 90° knee angle during the mid-drive phase (26). Therefore, squat power at 30% and 50% load may also reflect performance in the later leg drive phase, where force decreases but velocity and muscle activation rise.

While the results considering the power at high velocities in the squat were somewhat surprising the associations between the power at different intensities (30%, 50%, and 70%) of the deadlift 1 RM with 2,000 m rowing ergometer performance were more plausible. These associations were also observed for the different race phases (i.e., 500 m split times; see Table 4). Since the deadlift exercise might simulate the athlete's capability of connecting the leg drive with the finish phase, characterized by a change from sustaining high forces in the early part of the stroke to lower force but increasing velocities in the later part of the rowing stroke (22), an association with power at low and high loads (and thus a variety of MVs) is not surprising. Although a different measure, this would align with our previous findings on the importance of RFD measured over 300 ms in the isometric midthigh pull for explaining rowing performance in adolescent athletes (6). Given that boat velocity increases from the catch to the finish phase of the rowing stroke, while applied force further decreases (22, 27) the back and arm muscles (predominantly used in the bench pull) experience both high force at low velocity during the catch, and lower force at higher velocity later in the rowing stroke. These dynamics potentially explain the associations between 2,000 m rowing performance and power over the entire LVP in bench pull at 30%, 50%, 70%, 90% 1 RM for male athletes and 50%, 70% and 90% in female athletes. This trend was also evident in the sub-analysis of power at 30%, 50%, 70%, and 90% in relation to the corresponding four 500 m split times (see Tables 3 and 4).

In our previous study, we also showed that different phases of the rowing race (i.e., start, middle and final spurt) were associated with distinct neuromuscular abilities in adolescent rowing athletes (6). Thus, in addition to associations of maximal and explosive strength variables with the overall 2,000 m time, in the present study we also assessed 500 m splits. Since the 1 RM of all exercises and the PP of the deadlift and the bench pull were associated with the total 2,000 m time, it was not surprising that we also observed statistical associations with a considerable proportion of the analyzed 500 m split time (see Table 2). However, while the PP of squat was not statistically associated with the total 2,000 m rowing ergometer performance, we found a significant association with the first 500 m of a race. Rowing races typically follow a pacing strategy that emphasizes faster performance in the first and last 500 m, with a slower middle section (11). The associations observed may therefore suggest a greater reliance on leg drive during the initial 500 m, where high power output is needed to accelerate the boat from a standstill (3).

## 5 Study limitations

Several limitations should be acknowledged. Although all participants were well-trained athletes with at least one year of strength training experience, individual technical proficiency, particularly in complex exercises like the deep squat, may have influenced the ability to produce high movement velocities. Additionally, the athletes in this study may not have reached their full neuromuscular potential compared to peers at similar competitive levels (see Supplementary Table 2), which may affect the generalizability of the findings. While BMI was included as a covariate to account for differences in body composition across age groups and sexes, future research should consider more precise assessments (e.g., fat and lean mass) and markers of biological maturity. Moreover, although the analysis of discrete segments within a rowing race (i.e., 500 m split times) may yield valuable insights into specific neuromuscular demands, the resolution provided by these four segments may be insufficiently fine-grained. Consequently, the observed associations closely approximate those observed for the overall 2,000 m race time. To achieve a more precise understanding of segment-specific demands, it may be beneficial to analyze shorter intervals, such as the initial 10–20 strokes representing the start phase or the final 20 strokes corresponding to the finish phase.

## 6 Conclusion

Our findings highlight that in addition to the 1 RM in rowing-specific strength exercises, especially the PP of the deadlift and bench pull were associated with rowing ergometer performance. Thus, we conclude that besides maximal strength, the ability to generate high MVs might also add to the neuromuscular demands in rowing. This was supported by associations of the power at high movement velocities of squat (i.e., 30%, 50%), deadlift (i.e., 30%, 50%) and bench pull [i.e., 30%, 50% (male) and 50% (female)] and the 2,000 m rowing ergometer performance as well as the individual 500 m split times. However, potential differences in lower- vs. upper-body power and their specific associations with different phases of the rowing stroke require further investigation. Additionally, since this study only incorporated a cross-section design, future studies should assess whether chronic strength training focusing on maximal anticipated MVs contributes to improved rowing performance in these athletes.

## Data Availability

The raw data supporting the conclusions of this article will be made available by the authors, without undue reservation.
