# Supplementary material for: Individual load–velocity measures are associated with 2,000-m rowing ergometer performance in German national rowers
Source: Front Sports Act Living. 2025 Oct 7;7:1688650. doi: 10.3389/fspor.2025.1688650 (PMC12538512; doi:10.3389/fspor.2025.1688650)
Supplement: Supplementary file 1 [file Supplementaryfile1.docx]

Supplementary table 1: Strength measurements for male and female athletes, respectively. Data is presented as mean ± SD. The U17 category includes all athletes under the age of seventeen, the U19 category includes all athletes between the age of 17 and 19 years, the U23 category includes all athletes between the age 23 and 19 years and the Elite category includes all athletes older than 23 years.

| Male | | | | | | |
| --- | --- | --- | --- | --- | --- | --- |
|  | 1 RM | | | Peak power | | |
|  | Squat | Deadlift | Bench pull | Squat | Deadlift | Bench pull |
| U17  (n: 20) | 113.13 ± 22.46 | 126.00 ± 25.58 | 76.75 ± 9.63 | 469.44 ± 151.85 | 514.99 ± 121.10 | 448.74 ± 84.32 |
| U19  (n: 21) | 121.55 ± 17.93 | 135.36 ± 22.70 | 82.26 ± 8.66 | 518.05 ± 134.76 | 556.38 ± 115.52 | 499.32 ± 67.93 |
| U23  (n: 9) | 137.22 ± 17.70 | 165.56 ± 15.90 | 92.78 ± 6.78 | 594.79 ± 126.55 | 604.85 ± 142.81 | 574.13 ± 44.53 |
| Elite  (n: 13) | 139.64 ± 19.66 | 164.29 ± 26.08 | 103.32 ± 12.91 | 590.52 ± 135.96 | 664.90 ± 201.14 | 599.46 ± 103.39 |
| Female | | | | | | |
|  | 1 RM | | | Peak power | | |
|  | Squat | Deadlift | Bench pull | Squat | Deadlift | Bench pull |
| U17  (n: 10) | 73.50 ± 13.34 | 85.00 ± 17.48 | 49.55 ± 7.68 | 304.64 ± 101.09 | 362.32 ± 81.32 | 299.90 ± 61.16 |
| U19  (n: 14) | 81.07 ± 10.53 | 94.29 ± 12.82 | 55.57 ± 7.80 | 330.84 ± 73.38 | 396.78 ± 70.00 | 316.44 ± 43.83 |
| U23  (n: 20) | 90.63 ± 12.19 | 109.63 ± 15.69 | 61.25 ± 6.91 | 369.77 ± 77.69 | 417.02 ± 65.81 | 353.93 ± 47.48 |
| Elite  (n: 6) | 85.00 ± 14.49 | 101.25 ± 29.74 | 64.17 ± 8.16 | 346.61 ± 102.04 | 433.71 ± 87.66 | 376.92 ± 69.01 |


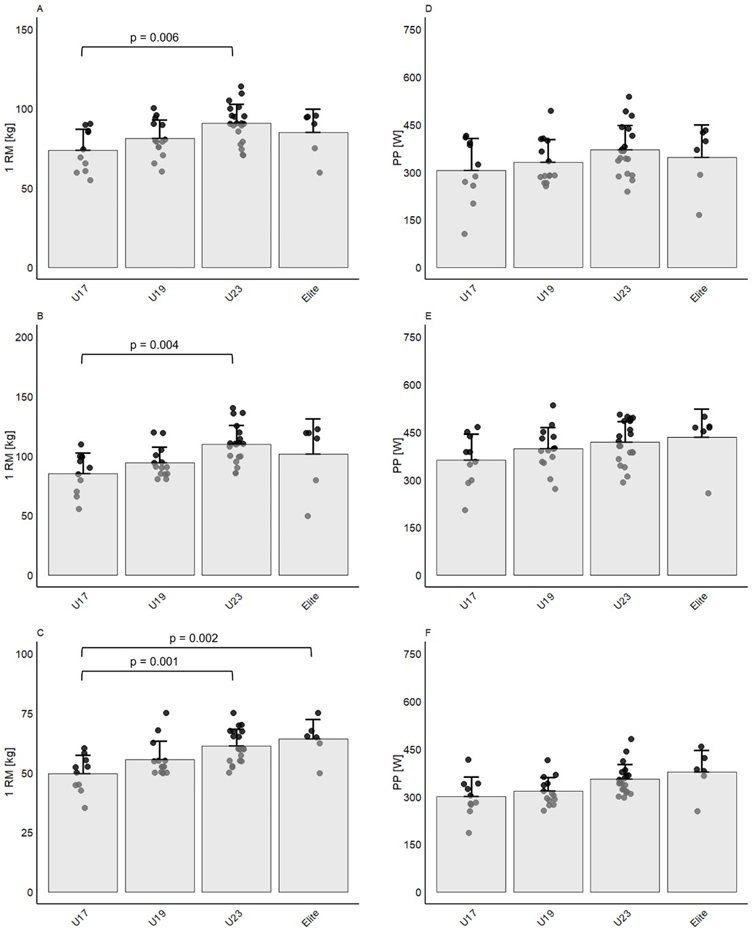


Figure 1: One repetition maximum (1 RM) of the squat (A), deadlift (B), bench pull (C) as well as and peak power (PP) of the three exercises (squat (D), deadlift (E), bench pull (F)) for different age groups (U17, U19, U23, Elite) in male athletes. The U17 category includes all athletes under 17, the U19 all athletes between 17 and 19 years, the U23 category all athletes between 23 and 19 years and the Elite category all athletes older than 23 years.


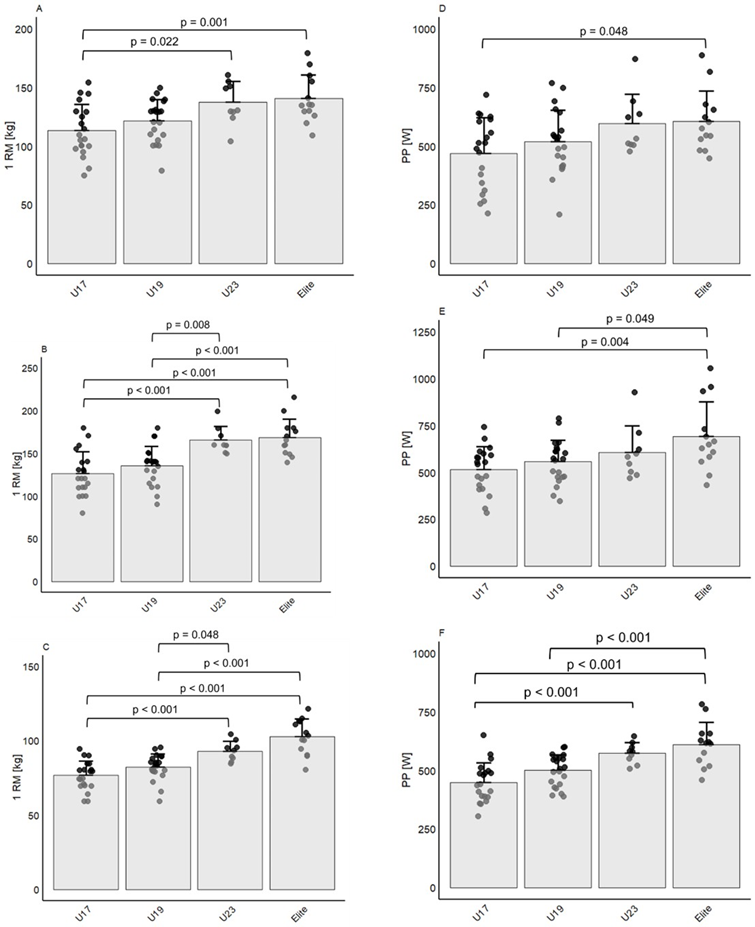


Figure 2: One repetition maximum (1 RM) of the squat (A), deadlift (B), bench pull (C) as well as and peak power (PP) of the three exercises (squat (D), deadlift (E), bench pull (F)) for different age groups (U17, U19, U23, Elite) in female athletes. The U17 category includes all athletes under 17, the U19 all athletes between 17 and 19 years, the U23 category all athletes between 23 and 19 years and the Elite category all athletes older than 23 years.

Supplementary table 2: Relative strength values of male and female athletes for the exercises squat, deadlift and benchpull. Reference values of (Santos Junior, Evaldo Rui T. et al. 2021) for intermediate and advanced strength trained athletes (range) as well as (McNeely et al. 2005) for strength goals of successful rowing athletes.

|  | Squat | Deadlift | Bench pull | Squat | | Deadlift | | Bench pull |
| --- | --- | --- | --- | --- | --- | --- | --- | --- |
|  | DRV Athletes | | | Santos Jr. et al. | McNeely et al. | Santos Jr et al. | McNeely et al. | McNeely et al. |
| Female Athletes | | | | | | | | |
| Elite | 1.13 | 1.34 | 0.85 | 1.0 – 1.3 | 1.6 | 1.2 – 1.6 | 1.6 | 1.2 |
| U23 | 1.18 | 1.43 | 0.80 | 1.0 – 1.3 | 1.4 | 1.2 – 1.6 | 1.4 | 1.1 |
| U19 | 1.02 | 1.29 | 0.76 | 1.0 – 1.3 | 1.25 | 1.2 – 1.6 | 1.25 | 0.95 |
| U17 | 1.10 | 1.27 | 0.74 | 1.0 – 1.3 | 1.0 | 1.2 – 1.6 | 0.8 | 0.6 |
| Male Athlete | | | | | | | | |
| Elite | 1.49 | 1.79 | 1.09 | 1.2 – 1.5 | 1.9 | 1.5 – 1.8 | 1.9 | 1.3 |
| U23 | 1.56 | 1.87 | 1.05 | 1.2 – 1.5 | 1.7 | 1.5 – 1.8 | 1.7 | 1.2 |
| U19 | 1.41 | 1.57 | 0.96 | 1.2 – 1.5 | 1.4 | 1.5 – 1.8 | 1.4 | 1.1 |
| U17 | 1.41 | 1.57 | 0.95 | 1.2 – 1.5 | 1.0 | 1.5 – 1.8 | 1.0 | 0.7 |
